# Effects of early life adverse experiences on the brain: implications from maternal separation models in rodents

**DOI:** 10.3389/fnins.2014.00166

**Published:** 2014-06-17

**Authors:** Mayumi Nishi, Noriko Horii-Hayashi, Takayo Sasagawa

**Affiliations:** Department of Anatomy and Cell Biology, Nara Medical UniversityKashihara, Japan

**Keywords:** maternal separation, HPA axis, depression, corticosteroid, gene expression, behavior, epigenetics

## Abstract

During postnatal development, adverse early life experiences affect the formation of neuronal networks and exert long-lasting effects on neural function. Many studies have shown that daily repeated maternal separation (MS), an animal model of early life stress, can regulate the hypothalamic-pituitary-adrenal axis (HPA axis) and affect subsequent brain function and behavior during adulthood. However, the molecular basis of the long-lasting effects of early life stress on brain function has not been fully elucidated. In this mini review, we present various cases of MS in rodents and illustrate the alterations in HPA axis activity by focusing on corticosterone (CORT). We then show a characterization of the brain regions affected by various patterns of MS, including repeated MS and single time MS at various stages before weaning, by investigating c-Fos expression. These CORT and c-Fos studies suggest that repeated early life stress may affect neuronal function in region- and temporal-specific manners, indicating a critical period for habituation to early life stress. Next, we introduce how early life stress can impact behavior, namely by inducing depression, anxiety or eating disorders, and alterations in gene expression in adult mice subjected to MS.

## Introduction

As our contemporary society changes rapidly, changes in family structure can have a large influence on the mother–child relationship, as well as on other social environmental factors. In adult patients with various neuropsychiatric disorders, childhood abuse including sexual and/or physical abuse and neglect, is one of the most serious causes (Bremne and Vermetten, 2001; Heim and Nemeroff, 2001; Teicher et al., 2006). Adverse experiences occurring during critical periods of development, such as perinatal life, harmfully influence behavior, and physiological functions, including growth, metabolism, reproduction, and immune responses. Stressful environments in early life may induce permanent rather than transient consequences in animals. Previous studies have indicated that early unfavorable events augment the risk of behavioral disorders in adulthood, including neuropsychiatric disorders, such as depression (Kendler et al., 2002) and psychosis (Morgan et al., 2007). In rodent and primate models, adverse environments during the neonatal periods seem to play a critical role in developing the brain systems important to regulate behavior and stress responsiveness. In particular, the responsiveness of the hypothalamic-pituitary-adrenal (HPA) axis can be deteriorated by interrupting usual mother-pup interactions, which may induce persistent changes in the neurobiology, physiology, and emotional behavior in adult animals (Ellenbroek et al., 1998; Lyons et al., 1998; Pryce et al., 2005; Enthoven et al., 2008; Nishi et al., 2013).

In this mini review, we will focus on the response of corticosterone (CORT), an end product of the HPA axis in rodents, and c-Fos expression for examining the activated brain regions induced by maternal separation (MS), a model of rodent early life stress. Furthermore, we will also present alterations of behavioral aspects and alterations in gene expression.

## Early MS

The inventive studies of Levine and colleagues, and consequently of Meaney, Plotsky, and their collaborators have demonstrated that changes in rodents' early postnatal experiences can induce profound long-lasting effects on emotionality and stress response (Levine, 1967; Meaney, 2001; Plotsky et al., 2005), which have spurred the employment of the rodent MS for investigating early life stress. This early life stress model is based on the evidence that unfavorable events in early life cause the vulnerability for developing various kinds of diseases in later life. In this type of study, MS should be carefully discussed in comparison to the appropriate control group, which may or may not be undisturbed from mother.

The procedure of MS showed a variety of the duration (e.g., 60 min–24 h) and the number of days (e.g., 1–14 days, 15–21 days) for the separation experiences among laboratories (Biagini et al., 1998; Caldji et al., 2000; Barreau et al., 2004; Arborelius and Eklund, 2007; Carrera et al., 2009; Tjong et al., 2010). In MS paradigm, many experiments, but certainly not all, have demonstrated that separation of pups from their mothers during the early postnatal period permanently increased anxiety-like behaviors in adulthood (Francis et al., 1999; Huot et al., 2001, 2004; Menard et al., 2004). As to the HPA axis activity, the response to stress is relatively low during early postnatal life (Walker et al., 1991; Levine, 2005), while MS could lead to life-long hyperactivity of the HPA axis (Holmes et al., 2005; Lippmann et al., 2007; Aisa et al., 2008; Marais et al., 2008). In contrast, short-term disturbance (e.g., 15 min), which has been called “handling,” appeared to reduce anxiety-like behaviors, decrease HPA axis tone and reduce the response to stress in adulthood (Levine, 2005; Plotsky et al., 2005). The process of handling may imitate natural mice rearing, whereby the mother leaves her pups for short periods of time to collect foods. Thus, the short-term MS, handling, might be considered a more natural event.

The effect of MS also varies depending upon whether pups are separated in a group of littermates during MS or isolated singly. Miyazaki and colleagues recently reported that rat pups isolated singly from the mother during PND7 to PND11 presented disturbance of cortical function, whereas pups separated but gathered from PND7 to PND11 showed no cortical disruption (Miyazaki et al., 2012).

## Characterization of maternally separated animals

### Serum level of CORT

**Figure 1:**
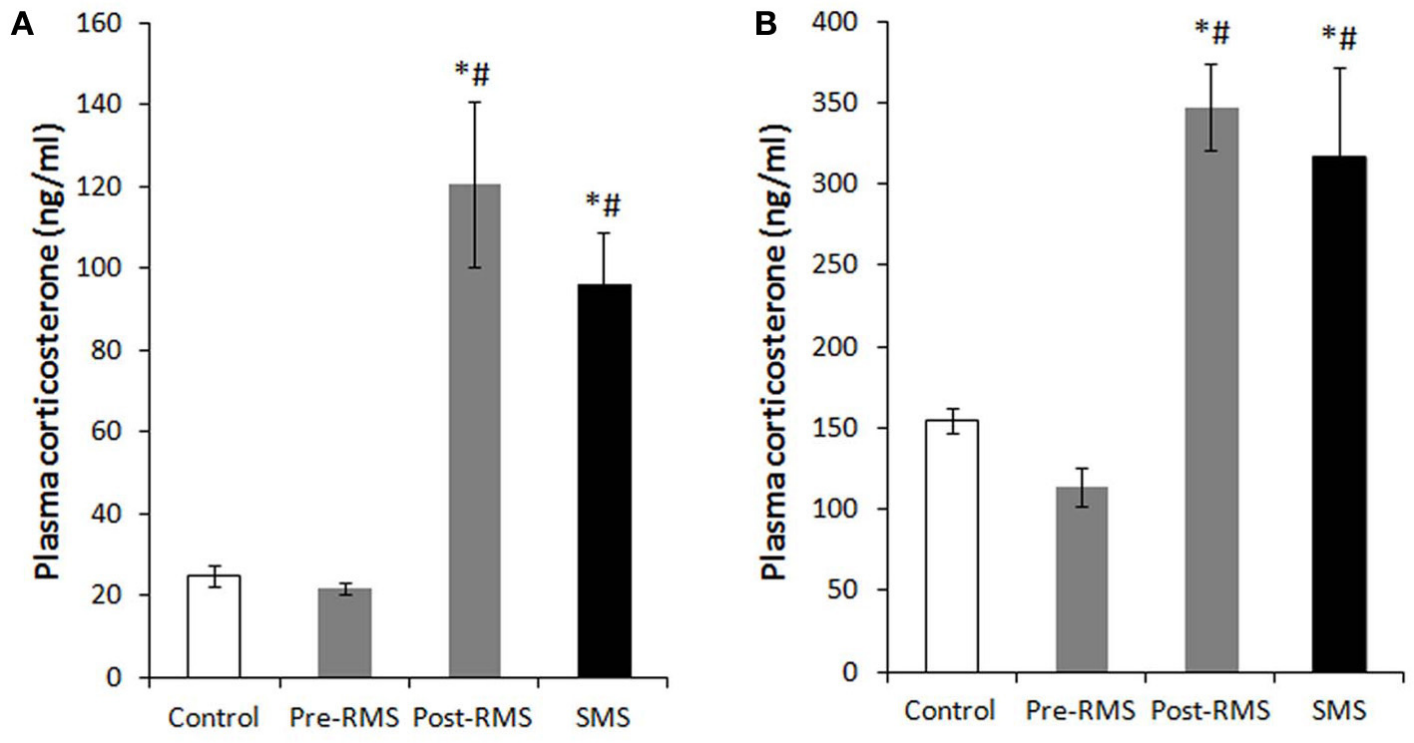
**Plasma CORT levels of repeated maternal separation (RMS) and single-time maternal separation (SMS) mice on PND14 and PND21 (Horii-Hayashi et al., 2013)**. The graphs show plasma CORT concentrations of PND14 **(A)** and PND21 **(B)** (*n* = 5–9 for each group). Blood samples were collected before (pre-RMS) and after (post-RMS) the final separation from RMS mice and after the separation from SMS mice. ^*^P < 0.05 vs. control, ^#^*P* < 0.05 vs. Pre-MS.

In rodents, there is an unique period during which the HPA axis shows a rapid regression known as the stress hyporesponsive period (SHRP) (Levine, 2001). This period extends from PND4 to PND14 in rats and from PND2 to PND12 in mice. During the course of SHRP, ACTH in increased and baseline plasma glucocorticoid levels are lower than normal (Rosenfeld et al., 1991). Because, during ontogeny, the maintenance of low and stable levels of CORT is necessary for normal growth and development of the central nervous system (CNS), the SHRP is hypothesized to be neuroprotective against stress-induced excessive stimulation of glucocorticoid receptors (GRs) (Sapolsky and Meaney, 1986; Sapolsky, 1996). In rodents, the presence of the mother appears to suppress HPA axis activity, which primarily preserves the SHRP. Indeed, even during the SHRP, MS is a compelling inducer of a stress response. Meaney and his colleagues suggest that the quality of the mother-pup interactions, such as increased maternal licking, grooming, and arched-back nursing, is an important aspect for the preservation of this dampened HPA axis activity (Francis et al., 1999). The disturbance of SHRP induced by MS could cause an excessive exposure of the brain to high concentrations of glucocorticoids and activation of GRs, which may subsequently regulate brain and behavior in later life. Enhanced secretion of stress-induced CORT was observed in pups separated from their mothers for 1 h on PND2 to PND9 (McCormick et al., 1998). Nevertheless, a recent study indicated that repeated MS for 8 h daily from PND3 to PND5 rapidly desensitized the HPA axis activity of neonatal mice (Enthoven et al., 2008). We also reported that repeated MS for 3 h daily from PND1 to PND14 did not elevate a baseline level of CORT on PND14, whereas a single-time MS for 3 h at PND14 raised a baseline CORT level (Figure 1) (Horii-Hayashi et al., 2013). In contrast to the effects of MS on neonatal animals, repeated MS for 3 h daily from PND1 to PND14 significantly raises a CORT level in adulthood, as reported by many studies (Ryu et al., 2008; Jahng et al., 2010; Horii-Hayashi et al., 2013).

### Activated brain regions analyzed by c-Fos expression

**Figure 2:**
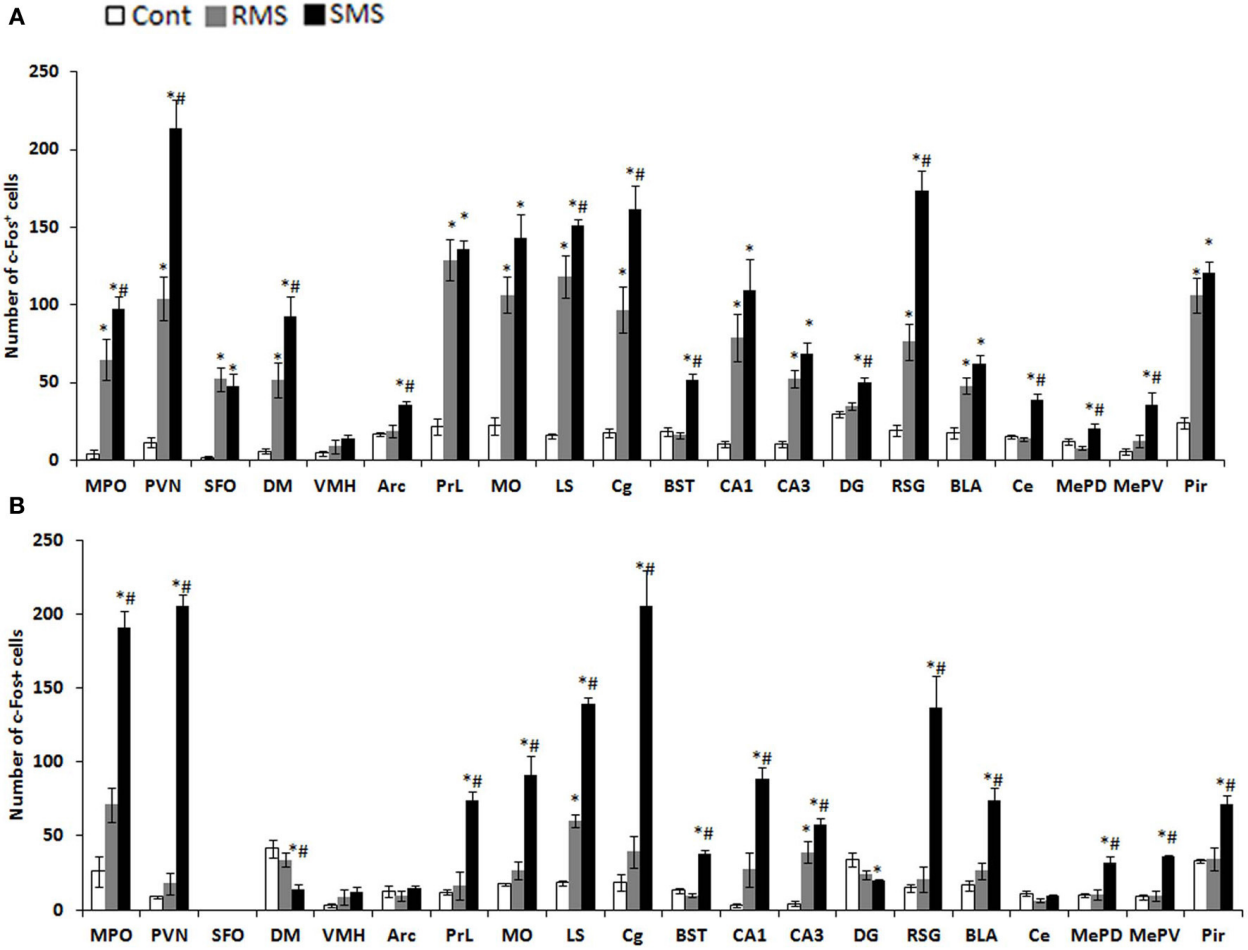
**c-Fos expression in the hypothalamus and limbic forebrain after MS (Horii-Hayashi et al., 2013)**. The graphs show the numbers of c-Fos-positive cells on PND14 **(A)** and PND21 **(B)** in non-separated control (white bar), RMS (gray bar), and SMS (black bar) mice (*n* = 4–5 for each group). In both RMS and SMS, the sampling point is just after MS procedure. ^*^P < 0.05 vs. control; ^#^*P* < 0.05 vs. RMS. MPO, medial preoptic area; PVN, paraventricular nucleus; SFO, subfornical organ; DM, dorsomedial hypothalamic nucleus; VMH, ventromedial hypothalamic nucleus; PrL, prelimbic cortex; MO, medial orbital cortex; LS, lateral septum; Cg, cingulate cortex; BST, bed nucleus of stria terminalis; CA1, hippocampal area CA1; CA3, hippocampal area CA3; DG, dentate gyrus; RSG, retrosplenial granular cortex; La, lateral amygdaloid nucleus; BLA, anterior part of the basolateral amygdaloid nucleus; Ce, central amygdaloid nucleus; MePD, posterodorsal part of the medial amygdaloid nucleus; MePV, posteroventral part of the medial amygdaloid nucleus; Pir, piriform cortex.

The expression of the immediate early gene product c-Fos is a reliable molecular marker to investigate neuronal activation. The examination of c-Fos expression has revealed that many brain regions are activated by MS, which differs depending on age and the type of stress. We recently analyzed the c-Fos expression induced by repeated MS and single-time MS during different developmental stages and time periods. Mice were exposed to 3 h repeated MS daily from PND1 to PND14 or from PND14 to PND21, or to single-time MS at PND14 or PND21 (Horii-Hayashi et al., 2013). We clarified that MS activated many brain regions and that c-Fos expression patterns changed developmentally (Figure 2). Single-time MS at both ages activated many regions of the hypothalamus and limbic forebrain, while the pattern of c-Fos expression in the repeated MS groups were significantly different on PND14 and PND21. In repeated MS of PND14 mice, the c-Fos expression levels in many regions were markedly increased compared with age-matched controls, excepting the VMH, Arc, BST, DG, Ce, MePV, and MePD. By contrast, in repeated MS on PND21 mice, c-Fos expression was reduced to control levels in all observed brain regions except for the LS and CA3. These findings suggest that repetition of a homotypic stimulus suppresses c-Fos expression by PND21, but that such suppression is barely observed on PND14. Moreover, in animals exposed to repeated homotypic stress during the postnatal period, increase in adrenal CORT secretion does not always associate with increased c-Fos expression in the PVN. Such developmental differences in c-Fos expression detected in the repeated MS groups may be associated with a developmental critical period for stress responses involving the HPA axis, during which animals are more susceptible to MS and other environments. In rodents, the critical period is the first two postnatal weeks. Thus, in early life, a repeated stress will be unlikely to suppress c-Fos expression. In turn, inappropriately activated c-Fos target genes may drastically alter how neurons function in critical neural circuits. Indeed, the suppression of increased c-Fos expression in repeated MS of PND14 mice was observed in specific regions (BST, Ce, MePD, and MePV) that form anatomical neural connections. These regions are referred to as an extended amygdala, which are closely associated with anxiety, fear, and psychiatric disorders (Davis et al., 2010). Therefore, even at PND14, repeated homotypic stress may reduce neural activity in the circuit of the extended amygdala. Moreover, in the SFO, where neurons are influenced by osmolality, calcium, and sodium concentrations in the systemic circulation (Smith and Ferguson, 2010), c-Fos expression was increased in both repeated and single-time MS mice, as compared to controls, on PND14. However, there were no changes in any of the groups on PND21. This difference may reflect the increased resistance of physical growth to the hyperosmolality induced by deprivation of lactation.

### Behavioral changes induced by MS in rodents

Early life adverse experiences including MS is one of the greatest contributing factors for mental health problems across life stages (Levine, 2005), relating not only to risk for mental health disorders but also to transdiagnostic features common in many psychological disorders (Glaser et al., 2006). I will introduce some of the behavioral aspects observed in animal model of MS.

#### Depression- and anxiety-like behaviors

Numerous studies have demonstrated a strong relationship between traumatic events during early life and development of behavioral abnormalities later in life. Early life adversity, such as that induced by MS, child physical, sexual, and emotional abuse, and general neglect has been linked to serious psychiatric impairment in adulthood (MacMillan et al., 2001). Particularly, a stressful life event such as early parental loss is associated with unipolar and bipolar depression, as well as anxiety disorders, beyond familial or genetic factors (Kendler et al., 1992; Agid et al., 1999; Furukawa et al., 1999; Heim and Nemeroff, 2001). Many human studies have reported that major depression and anxiety disorders are frequent in adults with a history of childhood abuse (Stein et al., 1996; Felitti et al., 1998). There have been numerous reports of the behavioral changes induced by MS in animal studies. Neonatal MS induces permanent alterations in the characteristics of the HPA response to stress in the offspring later in life (Ladd et al., 1996; Vazquez et al., 2000). Many studies of repeated MS during the first 2 weeks of neonatal life showed depression- and anxiety-like behaviors in adulthood (Newport et al., 2002; Daniels et al., 2004; Lee et al., 2007; Ryu et al., 2009). In these studies, ambulation and rearing decreased, immobility during a forced swim test increased, and time spent in the closed arms of an elevated plus maze increased.

#### Fear response

Until recently, no one had investigated how early experiences affected fear retention and extinction development, although these forms of emotional learning could be critically involved in the pathogenesis and treatment of mental health problems. Recent several studies showed that the timing of the maturation of fear learning is not set in static, but can be dynamically regulated by early experiences. Although the exact mechanisms are still unknown, when rats are reared under stressful conditions then they exhibit adult-like fear retention and extinction behaviors at an earlier stage of development (Callaghan et al., 2013). Chocyk et al. reported that MS decreased freezing time in both contextual and auditory fear conditioning in adolescent and adult rats (Chocyk et al., 2014). These results suggest that early life stress may permanently affect fear learning and memory.

#### Food intake and response to food deprivation

Previous studies showed that repeated MS during the first 2 weeks after birth may not permanently affect food intake and body weight gain of the offspring as long as the pups are reared in a group (Iwasaki et al., 2000; Kalinichev et al., 2002; Ryu et al., 2008). In contrast, post-weaning social isolation promotes food intake and weight gain of adolescent MS pups, with impacts on anxiety-like behaviors (Ryu et al., 2008). Anhedonia to palatable food, one of the major symptoms of depression, was reported in adolescent MS pups with disruption of the mesolimbic dopaminergic activity in response to stress (Noh et al., 2008). Another study showed that sustained hyperphagia observed in the MS pups subjected to a fasting/re-feeding cycle repeated during adolescent period of MS pups induced a binge-like eating disorder, in which increased activity of the HPA axis responding to such metabolic challenges appeared to play a role, at least partly, in mediation with the hypothalamic neuro peptide Y (NPY) (Jahng, 2011).

### Gene expression

Many animal studies, including MS, have improved our knowledge of gene-environment interactions and elucidated the pathways that program an animal in response to its early life experiences (Meaney and Szyf, 2005). Epigenetic mechanisms involving DNA methylation, post-translational modification of histone proteins and non-coding RNAs (most notably micro-RNA) are major candidates for regulating gene expression and integrating intrinsic and environmental signals in the genome (Jaenisch and Bird, 2003). Murgatroyd and colleagues showed that in the parvocellular subdivision of the paraventricular nucleus of the hypothalamus, MS in mice persistently upregulates *Avp* gene expression associated with reduced DNA methylation of a region in the *Avp* enhancer. This early life stress-responsive region serves as a binding site for the methyl-CpG binding protein 2, which in turn is regulated through neuronal activity. They also found that the ability of methyl-CpG binding protein 2 to control transcription of the *Avp* gene and induce DNA methylation occurred by recruiting components of the epigenetic machinery (Murgatroyd et al., 2009; Murgatroyd and Nephew, 2013). Other groups investigated DNA methylation levels at a specific sequence motif upstream of the GR gene (Nr3c1) in the hippocampus of offspring, and found that subjecting pups to a single 24 h MS increases methylation levels (Kember et al., 2012). The epigenetic alterations of these genes suggest that the HPA axis could be dysregulated by MS. Importantly, however, the DNA methylation differences were also often strain specific (Kember et al., 2012). Taken together, these findings demonstrate the importance of investigating environmental effects on a range of genetic backgrounds, emphasizing the need for the further examination of environmental, genetic, and epigenetic interactions.

## Conclusions

Adverse environments and experiences during the neonatal period can dramatically affect the development of the HPA axis that underlies adaptive behavioral responses. MS experiments, as a model of early life stress, demonstrate that CORT levels and c-Fos expression change depending upon the different experimental conditions of MS, e.g., age at testing and frequency of repetition. Furthermore, separation conditions (isolation with or without a littermate) could also influence the results of the MS experiments. MS can induce various behavioral changes manifested in later life, which could be caused, at least in part, by alterations in gene expression, particularly through epigenetic mechanisms.

### Conflict of interest statement

The authors declare that the research was conducted in the absence of any commercial or financial relationships that could be construed as a potential conflict of interest.
